# Triadic (ecological, neural, cognitive) niche construction: a scenario of human brain evolution extrapolating tool use and language from the control of reaching actions

**DOI:** 10.1098/rstb.2011.0190

**Published:** 2012-01-12

**Authors:** Atsushi Iriki, Miki Taoka

**Affiliations:** Laboratory for Symbolic Cognitive Development, RIKEN Brain Science Institute, 2-1 Hirosawa, Wako-shi, Saitama 351-0198, Japan

**Keywords:** primates, parietal cortex, spatial integration, coordinate transformation, non-spatial cognition

## Abstract

Hominin evolution has involved a continuous process of addition of new kinds of cognitive capacity, including those relating to manufacture and use of tools and to the establishment of linguistic faculties. The dramatic expansion of the brain that accompanied additions of new functional areas would have supported such continuous evolution. Extended brain functions would have driven rapid and drastic changes in the hominin ecological niche, which in turn demanded further brain resources to adapt to it. In this way, humans have constructed a novel niche in each of the ecological, cognitive and neural domains, whose interactions accelerated their individual evolution through a process of *triadic niche construction*. Human higher cognitive activity can therefore be viewed holistically as one component in a terrestrial ecosystem. The brain's functional characteristics seem to play a key role in this triadic interaction. We advance a speculative argument about the origins of its neurobiological mechanisms, as an extension (with wider scope) of the evolutionary principles of adaptive function in the animal nervous system. The brain mechanisms that subserve tool use may bridge the gap between gesture and language—the site of such integration seems to be the parietal and extending opercular cortices.

## 1. Introduction

In the course of human evolution and human history, our ancestors have created new habitats from modified hunter–gatherer environments to agricultural landscapes with villages, and then to modern civilized technological cities. The evolution of various new cognitive capacities, including those underwriting the manufacture and use of tools and the production and comprehension of languages, has enabled these ecological transformations. Such new cognitive capacities in turn are an outcome of the dramatic expansion of the human brain and of new functional brain areas. Humans have constructed a new ‘niche’ in each of these ecological, cognitive and neural domains. ‘Niche-construction’ denotes an evolutionary process whereby the activities of organisms modify their habitat, to which in turn the organisms evolve to adapt, thus creating their own ‘ecological niche’ in the environment [1–3]. This concept will be extended in this paper to include the ‘cognitive niche’ as a newly acquired class of cognitive capacity [4], and the ‘neural niche’ as a portion of neural tissue added through expansion of the brain [5,6].

The above three classes of niche have coevolutionary interdependencies. Such interactions might have accelerated hominin evolution, which seems remarkably rapid if it was simply the product of natural selection driven by exogenous environmental change. It is possible that ecological changes to habitats have occurred not as a cause of hominin cognitive evolution, but rather as a result of it, with consequent selection pressures acting on the neural basis of behavioural adaptations to the modified environment. New brain functions would constitute the basis for further innovation in cognitive functioning and thus further modifications to the ecological niche, providing a feedback loop for ‘triadic niche constructions’. In this paper, a potential evolutionary scenario that led humans to invent successively more complex forms of tools, and eventually to acquire the language faculty, will be proposed based on this dynamic interaction. The brain's functional characteristics play a key role in the above triadic interactions. The relevant neurobiological mechanisms are explored in this paper in order that the proposed evolutionary scenario should not be seen as teleological. Finally, we will try to locate these mechanisms as an extension of the evolution of the animal nervous system in general: human higher cognitive activity can then be viewed as continuous with that of other animal species comprising the wider terrestrial ecosystem.

## 2. Construction of the ecological niche

### (a) Hominin ecology structured through incorporating different classes of tools

Humans are peculiar, compared with non-human species, in the extent to which they try to ‘improve’ their habitual environment. To make such improvements, particularly in our modern urban environment, we make and use various kinds of tools and technologies, and often the tools themselves are incorporated into the fundamental structure of the environment to create a distinctive human ecological niche. For example, cars running on paved roads or air-conditioned skyscrapers are essentially artefacts in which to travel and to reside, yet also comprise an environment to which city inhabitants adapt both physically and perceptually. In earlier times, hominins may also have typically constructed their niche by gradually and consecutively incorporating artefacts into the habitual environment. Thus, one can ask whether the tools that comprise our environment can be classified and structured in hierarchical order and if so, how different modes of brain function subserve their use.

**Figure 1.**
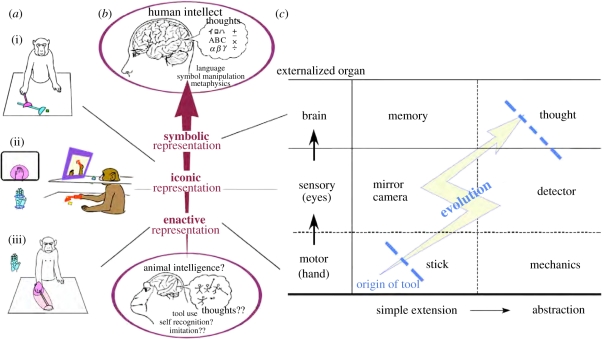
Various modes of cortical body-image codings (*a*) and hierarchical structure of various classes of tools (*c*) corresponding to the putative hierarchy of internal representations (*b*). (*a*(i)) Combinatory usage of short and long rakes. (*a*(ii)) When monkeys used a monitor, a visual receptive field of representative intraparietal bimodal neurons was formed around the hand in the monitor, encompassing its somatosensory receptive field. (*a*(iii)) When monkeys used a rake to retrieve distant food, the visual receptive field encompassed the somatosensory receptive field of a representative intraparietal bimodal neuron extended along the rake. Reproduced with permission from Iriki & Sakura [6].

The classical definition of the tool, as used in most existing tool-use studies, is restricted to external objects held by the hand and interacting with the external environments [7]. Namely, the tools so defined and studied are the ones that extend and externalize our hand, or more generally the motor organs or effectors. Indeed, the first series of tools that early hominins are known to have used [8,9], and those used by non-human animals [7] are these ‘motor tools’ (figure 1*c*, bottom row). We modern humans also use tools to extend or externalize our existing sensory organs, or to support the detection of information that is outside our natural sensory range (figure 1*c*, middle row). The optical telescope, endoscope or stethoscope would be examples of the former and the radio telescope or Geiger counter would be examples of the latter. Non-human animals rarely use this class of ‘sensory tools’ [10]. In monkeys, our own previous studies have demonstrated that they can be trained to use a sort of endoscope only after having acquired an ability to use a motor tool (a rake) [11]—as if attaching an additional visual cue to the tip of an extended body schema that was acquired through initial training to use a rake (see §2*b* for details). These results suggest that the class of sensory tools comprise a higher layer, superimposed onto previously acquired motor tools as the fundamental layer. Indeed, the history of our own technology suggests that sensory tools appeared much later, after motor tools were incorporated into human cultures.

What then would be the tool class of the third layer? If we looked at ourselves through our own externalized eyes (the second layer of the tools), we would observe ourselves as external objects by shifting from the first-person to the third-person perspective, in other words by ‘self-objectification’ [12,13]. This leads to the perception of our own intrinsically intransitive movement as transitive, i.e. to the acquisition of a sense of the self (as the subject), and leading to the movement of ourselves or our body parts perceived as objects. We may hypothesize that once the ‘self’ has been bifurcated into a subjective self and an objective self, the mind and/or intentionality emerges as a function that bridges those fragmented ‘selves’ and reunites them; this hypothesis has been proposed in detail elsewhere [6,13]. As a result of this self-objectification and emergence of the ‘mind’, a recognition of the ‘core self’ that continues across time from the past through the present towards the future may subsequently arise. Once the future self is recognized as having a core that is identical to that of the present self, one might wish to save the present information for future use. This can be accomplished by taking notes or drawing pictures, which requires an external device for memorizing facts; thus, an ‘externalization of the brain’ is produced as the tool class of the third layer (figure 1*c*, top row) [6,14].

How could the three different classes of tools outlined above be incorporated into humans' habitual environments? The scenario outlined proposes that successive layers of tools (motor extensions, sensory extensions and symbolic externalized memory) can be incorporated into the environment by building on the pre-existing acquisition and incorporation of tool classes of the immediately lower level. Thus, a positive feedback would have emerged between new brain functions and resulting modifications of the habitual environment. In the course of such positive feedback processes, a brain function emerged for the mind and for future-directed ‘intentionality’, after which the feedback became guided by human intentions (‘intentional niche construction’) [6].

### (b) Parietal plasticity when incorporating tools into the body schema

In using these tools, what kind of neural mechanisms and what modes of operation are employed, and what kinds of neural changes, if any, are induced upon acquisition of the ability to use tools? Our previous studies, as illustrated below, demonstrate one such example. Although Japanese macaques normally do not use tools in their natural habitat, two weeks of extensive training will enable these animals to use a hand-held rake to retrieve a distant food object located out of reach [15]. This training must imply the ability to reorganize the image of the body to one in which the rake is incorporated as an extension of the forearm. The body image is thought to form by integrating somatosensory and visual information relating to the body [16]. Thus, its modification after tool use could be physically observed as changes in the receptive field properties of the neurons that code such images [17]—when the tool was incorporated, the receptive field that codes the image of the hand was elongated to include the rake (figure 1*a*(iii)). This modification seemed to match the monkeys' internal states, whether or not the rake was incorporated into the image of the forearm. Here, an equivalence is established between body parts (hands) and tools, i.e. hands are extended towards tools (externalization of the innate body) or tools are assimilated into the body schema (internalization of external objects). Hence, these representations of the body image comprise an internal model of the bodily structures used to control various movements, as a concrete neural correlate of the ‘enactive representation’ [18], a class of representation that first emerges at a very early stage of postnatal development in human infants (figure 1*b*).

As the training proceeds further, we might postulate that the monkey's mode of representation may advance to ‘iconic (visual)’ and even close to ‘symbolic’ [18]—during human development, these appear later during childhood or after maturity. This expectation implies that motor-tool-use-trained monkeys could be further trained to use a video monitor to retrieve food that is out of their direct line of sight, and that the receptive field of the parietal neurons that code the hand and the tool incorporated into it will be activated by visual feedback when images of the hand and tool are seen on the video monitor (figure 1*a*(ii)) [19]. Thus, the body image is visually projected onto the distant monitor screen. In fact, we have found that monkeys that acquire the ability to use a rake and a video monitor to retrieve food objects in this way can immediately combine multiple tools purposefully to accomplish the goal [20], as if they are able to logically structure body parts using (proto-)symbolic representations (figure 1*a*(i)). Here, we can recognize that the hierarchical structures of motor tools/sensory tools/brain tools [18] resemble the hierarchy of representations from enactive (motor) to iconic (sensory) to symbolic (brain) structures of development, as described earlier (see figure 1*a*–*c* for these comparisons) [6]. Thus, reorganization of the modes of visuomotor integration in the parietal cortex must be crucial for the acquisition of these successively more advanced modes of representation.

## 3. Construction of the neural niche

### (a) Brain expansion by tool-use training

**Figure 2.**
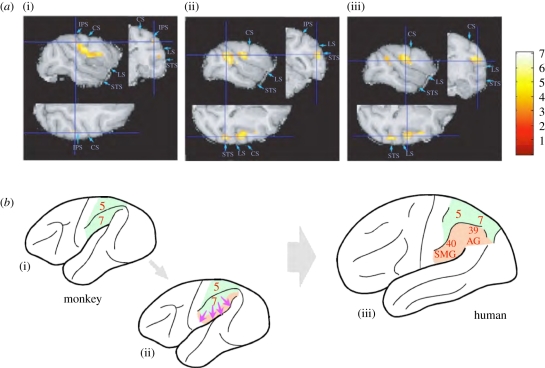
Grey matter increase with improvement in rake task performance. (*a*) Areas where grey matter increased with increasing performance score on the rake task. Sagittal, coronal and horizontal planes with increases in grey matter, including the right intraparietal sulcus (IPS, (i)), the superior temporal sulcus (STS, (ii)) and the secondary somatosensory area (SII, (iii)), are shown. (*b*) Schematic illustrating how tool-use-induced expansion of the parietal cortex of monkeys (i,ii) may contribute to the establishment of a precursor for the formation of human inferior parietal areas (iii), thus creating a novel neural niche that subserves further higher cognitive functions. CS, central sulcus; LS, lateral sulcus. The colour scale indicates the *t* score. (*a*) Reproduced with permission from Quallo *et al*. [21].

Is the neural plasticity depicted above limited within the range of the individual's learning capacity, or could it cumulatively evolve over generations? In other words, is it purely subserved by ‘cultural inheritance’, or alternatively, could it be a part of an epigenetic evolutionary mechanism in which the information embedded in the environment contributes to modification of phenotypic expression in succeeding generations? Although the latter has a flavour of Lamarckism—inheritance of acquired phenotypic traits—there may be a biological mechanism that could channel the evolution of adaptations to an environment in which cultural information is embedded. Macroscopic expansion (up to 23% of MRI grey matter signal) of cortical grey matter, including the intraparietal region, was detected in monkeys undergoing two weeks of tool-use training (figure 2*a*) in our recent Voxel Based Morphometry analysis (a kind of digital neuroanatomy using the magnetic resonance imaging technique) [21]. During the same period, microscopic changes (axonogenesis and synaptogenesis, as detected by tracer-injection histological examinations) [22] together with elevated expression of immediate-early genes [23] and of neurotrophic factors [24,25] were also shown to have been induced in these cortical areas. The grey matter expansion extended to include adjacent areas, such as the secondary somatosensory area (figure 2*a*(iii)) and the surrounding opercular cortex. Although the evidence obtained to date remains fragmentary and more detailed biological examinations are in progress, these initial findings indicate that the brain is much more adaptive than was previously believed: exposure to a novel cultural environment induces the brain to exhibit not only functional plasticity, but also extensive and persistent morphological change.

This implies that once a novel cognitive demand, such as incorporation of motor tools into the body schema, has become embedded in the environment, modifications of brain structure would be induced automatically through the normal developmental processes in succeeding generations. The occurrence of such a plastic response during the lifespan as a result of behavioural modifications that lie within the existing adaptive capacity of individuals, and its subsequent consolidation (under selection acting on changing gene frequencies) as a default state that is stable over generations [26–28] is termed the ‘Baldwin effect’ [29,30], and comprises one potential component of the evolutionary process.

### (b) Redundant and polysemic systems as pre-adaptations for a novel neural niche

What could be the biological principles that allow brain expansion as an evolutionary mechanism of the kind just outlined? Biological systems are never ultimately efficient—systems require some redundancy for stability, to avoid over-specialization that might threaten the capacity to survive new challenges. Some redundancy in brain structure would allow representational bistability, for both the originally adapted functions and functional response to the new challenge. Increased redundancy to stabilize this functional capacity for flexible adaptive responses, perhaps via rapid brain expansion, would also then allow rapid construction of new and specialized neural resources. As has been discussed earlier, monkey intraparietal neurons that normally code body image can be trained to code a tool in a way that is equivalent to that for the hand holding it [17]. Thus, these neurons are bistable or polysemous for the hand or the tool. This functional plasticity may be an inherent property at the margins of a neural coding system prepared for gradual elongation of the arm during body growth, and which can then also adapt to a ‘sudden elongation’ by using the tool. This accidentally established equivalence between body parts (hands) and tools in turn leads to additional polysemic and bistable *interpretations*, i.e. hands may be extended into the tool representation (externalization of the innate body) or tools may be assimilated into the body schema (internalization of external objects).

Thus, redundancy in the brain, initially adapted to stabilize this system against unexpected environmental noise (or developmental changes, following the above speculation about body growth) has occasionally allowed the system to be polysemous. This newly acquired bistable state enables the reuse of cortical systems for different functions in the future, as in the case of tool use, perhaps in combination with other parts of the brain [5,31]. This bistability, or ‘polysemy’, could enable the use of metaphors in conceptual structure—so as to comprise a novel cognitive niche, as will be described in the next section. However, how human higher cognitive functions appear to have ‘evolved’ much more quickly than might be expected from ordinary biological evolutionary processes of adaptation to changing external environmental contingencies remains an open question. Humans can induce such changes intentionally, to construct a better-fitting ecological niche [6]. The capacity of human intention, or of the human mind, to plan for the future emerged through the process described in the previous section in relation to the hierarchy of classes of tool use (motor, sensory and brain). Subsequently, the neural systems which process the information that is necessary to inhabit the tool-modified environment (the neural niche of the brain; figure 2*b*(ii)) could be reinforced further by extragenetic or epigenetic triggering factors embedded in such an environment.

In addition, because hominin species have attained an unusually long post-reproductive lifespan, particularly females [32], accumulation of knowledge continues over the whole lifespan of an individual, tending to peak in middle-to-old age. Thus, some extragenetic mechanisms are indispensable for inheritance of these later acquired ‘cognitive niches’ over generations to occur. Species with a short lifespan and mass reproduction adapt to environmental changes through variations in their numerous offspring, as they expect that at least a few will survive, whereas species with a long lifespan—such as primates, and most typically humans—and low birth rate do so through an individual capacity to adapt [33]. This process would be enhanced by expansion of an organ that controls adaptive behaviours, namely the brain, which are stabilized as the typical phenotype of the species through epigenetic mechanisms. The evolutionary process driving such expansion may not simply be natural selection acting on random mutations, but rather something like the Baldwin effect [29,30] as depicted earlier.

## 4. Construction of the cognitive niche

### (a) Parietal ‘neural niches’ for processing spatial and non-spatial cognition

Debate exists on the comparative anatomy of the primate parietal cortex (figure 2*b*). One view claims that the inferior parietal area is evolutionarily new and uniquely expanded in humans, and that therefore the monkey posterior parietal cortex corresponds to the human superior parietal lobule. This is the position illustrated in the scheme presented in figure 2*b*(iii), independent of figure 2*b*(i) [34,35]. Evidence supporting this view would include the fact that, for example, the superior parietal lobule tends to process spatial information in a conventional way, whereas the inferior parietal lobule is often credited with non-spatial cognition. Many recent human imaging studies demonstrate that this cortical area additionally supports various forms of high-order non-spatial cognition that are not necessarily directly related to physical space itself. Indeed, a comparison of the brain areas responsible for tool-use behaviours in monkeys and humans [36] detected a patch of the parietal cortex specific to humans that might be responsible for perception and manipulation of abstract causal relationships required for human tool-use behaviours. This could be evidence of a function that was derived from a polysemic mechanism as described above, in which an additional resource of brain tissue (a neural niche) functions to enable an additional cognitive process (a cognitive niche).

**Figure 3.**
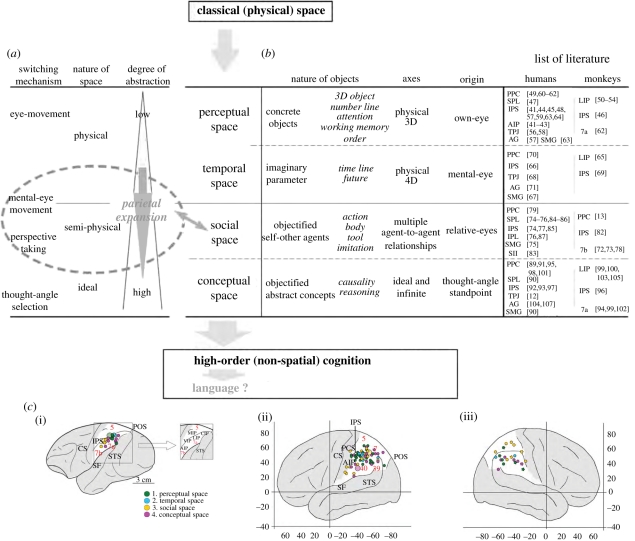
(*Caption opposite.*)

However, there is another view in which the monkey parietal cortex includes functional homologues of both regions [37–40], which allows viewing figure 2*b*(i–iii) as a continuum. Various kinds of non-spatial cognition can be grouped and ordered based on the levels of abstraction of the ‘objects’ and the conceptually defined spaces which are represented. The assumed coordinate systems for such ‘spaces’, with citations to research literature analysing the brain mechanisms which encode such spatial coordinates (for monkeys and humans), are summarized in figure 3 [108]. The pseudo-spatial nature of the high-order cognition supported by the posterior parietal cortex may be derived from the essential characteristics of the objects represented; alternatively, it may be derived from the nature of the pre-existing information-processing mechanisms of this area, namely as a hub for multi-sensory integration and for representing physical environmental space [109]. A meta-analysis of the references listed in figure 3 (refer to its legend for a detailed classification of various cognitive niches handled by this brain area) shows that the posterior parietal areas responsible for these novel forms of cognition are not necessarily clearly segregated, either in monkeys or in humans, and suggests a trend of gradual expansion towards the lateral sulcus as the level of abstraction increases (figure 3*c*) [108]. Thus, it seems that the parietal area gradually incorporated high-order cognition as it expanded during hominid evolution, while preserving its original principles of operation. This could be an example of exhibiting a novel cognitive niche by re-using the functions that have derived from a polysemic mechanism described previously. If this was the case, the gradual emergence and differentiation of functions in the transitional state might not be detected until the intensity of activation and the quantity of tissue recruited to serve this new neural niche exceeded some threshold of detection. In this sense, the above two views of parietal evolution are not necessarily mutually exclusive.

What, then, is the explanation for this series of additions of novel cognitive niches? Mechanisms for ‘selecting’ and ‘switching’ between objects among different represented spaces (figure 3*a*) could be hypothesized to contribute to this process [108]. That is, initially in classical (physical) space, spatial attention towards concrete objects was typically expressed as the direction of the eye axis to represent ‘perceptual space’ ([41–64]; see legend of figure 3 for details of respective references, and also for other classes of ‘spaces’ described below). Secondarily, when such attention had to be sustained or the attending content had to be memorized, invisible ‘time’ was ‘visualized’ in the mind's eye, becoming a new virtual dimension in the existing suite of spatial-coordinate systems, namely ‘temporal space’ [65–71]. And then, once one was able to visualize an invisible virtual entity, a similar objectification process could have been extended further, enabling intentional perspective switching. Acquiring representations of ‘social space’ [13,72–87] might have accelerated this process. Via self-objectification processes [13] mentioned in the earlier section, and the development of ‘virtual eyes’ [11,12], flexible and mutually integrated representations of the bodily self, of the analogous selves of others, and of tools used as equivalents of body parts (and vice versa) may have served as a bridge between concrete physical and abstract conceptual spaces. Finally, as the posterior parietal cortex expanded in both physical volume and in range of function [6,21], a positive feedback process could have been established to achieve further human-specific forms of non-spatial conceptual cognition, or ‘conceptual space’ [12,80,81,88–107]. In this way, crucial components of human intelligence would derive their character from the precursory spatial cognition process of the parietal cortex. Language is full of spatial metaphors for abstract thoughts.

### (b) Opercular cortex as a cradle for language by re-using spatial processing principles

During evolution, whenever organisms are faced with a novel and unforeseen environment, they have no other means to overcome immediate problems than to reuse any materials at hand [5,31]. Thus, cognitive capacities are extended by diverting pre-existing functions. In hominin evolution, according to the scenario outlined in the previous section, the expanded inferior parietal area and surrounding opercular region have taken on distinctive functional characteristics. Basic continuity from monkeys to humans as described above seems to be present in this general area, which includes Broca's area (anterior operculum), Wernicke's area (posterior operculum) and the middle operculum corresponding to the supramarginal and angular gyri, and which appear to be an extension of the inferior parietal lobule of the monkey brain; the continuity underwrites opercular language representations. Fundamentally, the argument from continuity implies that such representational capacity should be a simple extension of a coding system for reaching and grasping. Initially, this extension would derive from the coding of spatial integration and of a reorganized representation of space, which could be extrapolated further using a principle identical to the non-spatial higher-order coding of more abstract objects [108,110]. In particular, this extrapolation could be subserved by the ‘abstraction’ of free and unconstrained polysemous handling of the space and of the body, which would comprise a fundamental component of language representations, and perhaps also by common neural mechanisms that share a mode of information integration and processing.

Human-specific illogical cognitive biases for symmetrical inference (the tendency to incorrectly infer ‘if B then A’ from a conditional relationship ‘if A then B’) and for inference by exclusion (the tendency spontaneously to assume that an unfamiliar label goes with an unfamiliar object) involve these same brain areas [111,112]. The mind, human language and human cultural transmission, all of which contribute to the semantic inheritance of the benefits acquired during the unusually elongated human post-reproductive lifespan, are aspects of cognitive functions that have evolved recently and result from such neural niche construction. Once a fundamental syntactic SVO (subject/verb/object) structure emerges [113] and is generalized, abstraction and concept formation and their manipulation become possible and constitute a basis for further intellectual advancements, such as polysemic interpretation of phenomena (which enables metaphorical inference). Such redundancy and polysemous representation would allow equivalence and symmetric inferences and would lead to the emergence of symbols. All of these functions seem to be carried out in the expanded inferior parietal and surrounding areas in humans. Hence, the human language faculty seems to draw on these fundamental neural mechanisms, which are found in these late-myelinating brain areas, which retain a large degree of flexibility until adulthood.

## 5. Parietal cortex as the centre of triadic interactions

### (a) A site for multiple sensory and motor integrations and coordinate transformations

The posterior parietal cortex plays a central role in multi-sensory integration and recognition of environmental space. Such integration provides a basis for the production of movements of various body parts, including eyes, hand-arm, head and whole body through transformations between different coordinate systems. The principle of neural reuse [5,31], as depicted above, seems to apply here in enabling the evolution of higher cognitive functions and thus of human cultural niches. Once these fundamentals were established in the parietal cortex, prefrontal cortex could have developed further so as to use the information for further executive functions involving working memory and syntactic operations, which are often argued to have been crucial for the evolution of human intelligence [114]. How, then, could this function have been extrapolated from the general evolution of the nervous system? In this section, we shall sketch a possible sequence of grades of gradually increasing complexity that might take us from reaching movements to tool use and language.

**Figure 4.**
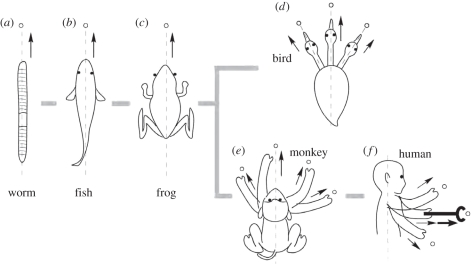
Patterns of reaching-and-grasping movements in different animal species. Fundamentally, animals reach and grasp by moving towards the targets (open circles) using their whole body (*a*–*c*). Higher vertebrates developed additional organs, elongated from the trunk to reach and grasp (birds (*d*, neck) and primates (*e*, forearm)). (*f*) In humans, the direction of the movements became perpendicular to the axis of the body because of bipedalism, and was further extended or transformed by the use of tools. Note the differences among the different species in the relative location of the eyes (and of the vestibule) relative to the organs used to finally reach and grasp, as well as their movements relative to the trunk and to the axis of the body (dashed lines).

Throughout evolution from primitive protozoa to mammals, the mouth was the organ used both to grasp and to intake prey, after reaching it through locomotion along the body axis. Some animals, especially primates, finally developed the hand to reach and grasp. The target to reach (prey) is detected by sensory organs of various modalities. The nervous system links these sensory and motor apparatuses to produce appropriate actions. The site of such integration within the neuraxis expanded continuously, to finally form the parietal cortex in primates; and its further extrapolation enabled the use of tools, as depicted above. The emergence of bipedalism constituted another evolutionary path for such expansion, as it differentiated the body axis from the movement axis, thus demanding a dramatic increase in spatial information transformation. In turn, this drove the evolution of the parietal operculum in the neural niche. Figure 4 illustrates this scenario, of which fundamental behavioural and neural correlates are summarized below.

#### (i) Head reaching

Primitive animals, in which the locomotor apparatus (such as fins or limbs) has not yet evolved, ‘crawl’ with the whole body to prey (figure 4*a*). The ‘mouth’ is located at the front end of the body axis [115,116], where sensory organs cluster to efficiently acquire environmental information. Moving in the direction of the mouth (i.e. ‘head reaching’) is still common in extant taxa, including vertebrates. Fish swim in the water three-dimensionally along the body axis (figure 4*b*). Terrestrial amphibians (and essentially also reptiles and most mammalian species) were constrained to move two-dimensionally on the land surface, yet they still move in the direction of the main body axis and crawl to reach, having evolved limbs for locomotion and resistance against gravity (figure 4*c*). Head reaching requires information on the target from a self-centred perspective. Animals align the body axis (the direction to move) towards the object and then approach it by travelling with the whole body until arriving at the target. The neural machinery used need only be the rather stereotyped projection of the body onto the environmental space, which requires minimal resources of neural tissue, and of which even insects' tiny brains are capable [117,118].

#### (ii) Neck reaching

Avians further developed, from forelimbs, the wings to fly. After a flight to reach prey, two final precise reaching-and-grasping procedures emerged. Raptors grasp the prey object by a hind limb [119] and finally eat it using the mouth/beak—they use organs other than the mouth just to grasp, but not directly to eat. Species with long flexible necks, like herons or cranes, reach and grasp directly with the beak using neck movements [120,121]. Here, a discrepancy emerges between the axes of the body and the head (figure 4*d*). As long as the mouth and eyes remain relatively fixed, the neural processing to reach with the mouth/head remains the same. But, once the neck can move independently of the trunk, object locations need to be represented in multiple spatial-coordinate systems—not only for the original coordinates with the body/trunk at the origin, but also with the head moving relative to the body. Such transformations between coordinate systems would have required their brains to evolve further neural resources.

#### (iii) Arm reaching

In mammals, particularly primates, the forelimbs have evolved as apparatuses to reach and grasp, diverging from their original locomotor function. Such evolution occurred via (i) substantial elongation of the forelimbs; (ii) increased degrees of freedom of movement at the shoulder, elbow and wrist joints; and (iii) elongation of digits to grasp objects of various sizes, shapes and orientations [122–124]. These changes dramatically increased the diversity of kinds and orientations of reaching-and-grasping motions in the space around the body axis (figure 4*e*). However, as a trade-off, it requires complex information processing by the brain to harmonize the movement of different body parts by translating positional information between different coordinate systems—body-centred, eye–head-centred and hand-centred systems [125]. Such situations demand more neural resources and the evolution of highly developed spatial perception, resulting in the expansion of parietal cortex. This was a cradle for the further evolution of transformations and modifications between coordinate systems, even for other working spaces. These served as a preadaptation by increasing the degrees of freedom for spatial information acquisition, thereby enabling further expansion of the brain areas that are responsible for those calculations.

#### (iv) Rotation of moving axis

These processes could be immediately extrapolated onto further evolutionary events. One of those would be the emergence of constant bipedalism. This consists in the maintenance of an upright head-lifted posture, with locomotion perpendicular to the axis of the body trunk for the first time in evolution (figure 4*f*). Additional constraints emerged, i.e. visual axes became fixed to the direction of locomotion (horizontal), thus also perpendicular to the axis of the body. As a result, various axes (body, hand, head and eyes) became dissociated and the directions of locomotion and of reaching/grasping became independent, depending on the ongoing behaviour. The brain mechanisms for processing such information remain incompletely understood, and open for future investigations.

### (b) Extension of axes from concrete to virtual spaces for locomotion, tool use and language

In evolution, the parietal cortex expanded initially as an adaptation to demands from the environment, perhaps for control of different movements of the various body parts, while prefrontal cortex may have expanded later to control such information coded in the parietal cortex through prefronto–parietal interactions [114]. In this way, neural mechanisms became embedded in the brain which served as pre-adaptations for further neural evolution through neural reuse, ultimately enabling the language faculties and (via prefrontal expansion) advanced modes of executive control. The emergence of bipedalism, through its associated demand on multi-sensory integration and very complex sensorimotor coordinate transformations, also pressurized expansion of the greater opercular regions of the cerebral cortex, and thereby facilitated subsequent reuse of such structures for higher cognitive functions including language.

Neural evolution in the first stage of this extension of axes put in place conditions for the emergence of tool use (derived from the usage of innate organs for a purpose not originally planned for). This polysemous pattern of organ usage, and the discovery of novel types of usage, would be key elements in inducing the re-evaluation of existing spatial structures in relation to the body axis as a second stage. The emergence of bipedalism triggered further extrapolation of such faculties and initiated the usage of the externalized body, i.e. extension of body parts into the tool representation. Such freedom from existing physical and bodily constraints in the understanding of the environmental space would allow a novel mode of spatial perception using novel tools (perspective transformation) that would be the basis for the next jump in the acquisition of abstract and transcendental thoughts—stage three. This development has served, in a final stage, as a cradle for the language faculty, principally by developing its neural basis for information processing, both for the use of polysemous and conceptual thoughts and for the articulation of oro-facial organs, to finally subserve language.

## 6. Conclusion

Expansion (or increase in capacity) of organs as an adaptive response to ecological pressures seems to be a general biological and evolutionary tendency to make the phenotypic system robust—the brain will not be an exception. Multi-sensory integration and coordinate transformation for the control of reaching movements in the inhabited space is an essential function of the nervous system, for which evolution finally endowed primates with a well-developed parietal cortex. The shift of body-space structure associated with the emergence of hominin bipedalism may have further pushed this trend forward to give this area, and the extended opercular cortex, further resources. Such neural enhancement (construction of the neural niche) happened to enable the processing of abstract information, detached from actual physical constraint, by applying and re-using existing principles for spatial information processing to realize novel mental functions (construction of the cognitive niche)—ultimately leading to language. Purposeful manipulation of the body image in space, required for tool use, would have accelerated interactive links between the neural and cognitive niches—tool use requires transformation of various bodily and spatial coordinates, as well as logical and sequential relations of action components.

Tools represent materialized cognitive brain functions. They have been created one after another and incorporated into hominin habitats as constituent elements (construction of the ecological niche). A human-modified environment puts pressure on succeeding generations to adapt to it, perhaps by acquiring further resources for the relevant organs. Epigenetically induced plasticity (including developmental or learning mechanisms) would participate in such processes—and this is a subject for future biological investigations. In this way, extra genomic information could be transmitted between generations via mutual interactions among ecological, neural and cognitive domains of niches, which may have contributed to hominin evolutionary processes (that is, ‘triadic niche construction’). This scenario would locate the human brain as part of an evolving holistic ecosystem.
